# Cardiopulmonary Resuscitation of Asystolic Newborn Lambs Prior to Umbilical Cord Clamping; the Timing of Cord Clamping Matters!

**DOI:** 10.3389/fphys.2020.00902

**Published:** 2020-07-30

**Authors:** Graeme R. Polglase, Georg M. Schmölzer, Calum T. Roberts, Douglas A. Blank, Shiraz Badurdeen, Kelly J. Crossley, Suzanne L. Miller, Vanesa Stojanovska, Robert Galinsky, Martin Kluckow, Andrew W. Gill, Stuart B. Hooper

**Affiliations:** ^1^ The Ritchie Centre, Hudson Institute of Medical Research, Clayton, VIC, Australia; ^2^ Department of Obstetrics and Gynaecology, Monash University, Melbourne, VIC, Australia; ^3^ Centre for the Studies of Asphyxia and Resuscitation, Neonatal Research Unit, Royal Alexandra Hospital, Edmonton, AB, Canada; ^4^ Department of Paediatrics, Monash University, Melbourne, VIC, Australia; ^5^ Newborn Research Centre, The Royal Women’s Hospital, Melbourne, VIC, Australia; ^6^ Department of Neonatology, Royal North Shore Hospital, The University of Sydney, Sydney, NSW, Australia; ^7^ Centre for Neonatal Research and Education, The University of Western Australia, Subiaco, WA, Australia

**Keywords:** delayed cord clamping, immediate cord clamping, asphyxia, chest compressions, asystole, epinephrine

## Abstract

**Background:** Current guidelines recommend immediate umbilical cord clamping (UCC) for newborns requiring chest compressions (CCs). Physiological-based cord clamping (PBCC), defined as delaying UCC until after lung aeration, has advantages over immediate UCC in mildly asphyxiated newborns, but its efficacy in asystolic newborns requiring CC is unknown. The aim of this study was to compare the cardiovascular response to CCs given prior to or after UCC in asystolic near-term lambs.

**Methods:** Umbilical, carotid, pulmonary, and femoral arterial flows and pressures as well as systemic and cerebral oxygenation were measured in near-term sheep fetuses [139 ± 2 (SD) days gestation]. Fetal asphyxia was induced until asystole ensued, whereupon lambs received ventilation and CC before (PBCC; *n* = 16) or after (*n* = 12) UCC. Epinephrine was administered 1 min after ventilation onset and in 3-min intervals thereafter. The PBCC group was further separated into UCC at either 1 min (PBCC_1_, *n* = 8) or 10 min (PBCC_10_, *n* = 8) after return of spontaneous circulation (ROSC). Lambs were maintained for a further 30 min after ROSC.

**Results:** The duration of CCs received and number of epinephrine doses required to obtain ROSC were similar between groups. After ROSC, we found no physiological benefits if UCC was delayed for 1 min compared to immediate cord clamping (ICC). However, if UCC was delayed for 10 min after ROSC, we found significant reductions in post-asphyxial rebound hypertension, cerebral blood flow, and cerebral oxygenation. The prevention of the post-asphyxial rebound hypertension in the PBCC_10_ group occurred due to the contribution of the placental circulation to a low peripheral resistance. As a result, left and right ventricular outputs continued to perfuse the placenta and were evidenced by reduced mean pulmonary blood flow, persistence of right-to-left shunting across the ductus arteriosus, and persistence of umbilical arterial and venous blood flows.

**Conclusion:** It is possible to obtain ROSC after CC while the umbilical cord remains intact. There were no adverse effects of PBCC compared to ICC; however, the physiological changes observed after ROSC in the ICC and early PBCC groups may result in additional cerebral injury. Prolonging UCC after ROSC may provide significant physiological benefits that may reduce the risk of harm to the cerebral circulation.

## Introduction

Perinatal asphyxia is the fifth most common cause of death in children <5 years of age (Saugstad, 2010) and is responsible for ~1 million deaths annually (23% of neonatal deaths; Duran et al., 2008; Lawn et al., 2011). Of the infants who survive, ~1 million develop adverse sequelae (Wall et al., 2010), which most commonly manifests as long-term neurological impairment (Lawn et al., 2007) that accounts for ~10% of all cerebral palsy cases (Saugstad, 2010). Thus, perinatal asphyxia is an important contributor to neonatal mortality and to major ongoing morbidity. In the most extreme cases of asphyxia, the infant is born atonic, apneic, and severely bradycardic or asystolic. In these infants, adequate ventilation alone is not sufficient to restore cardiac function. Current clinical guidelines recommend chest compressions (CCs) and adrenalin administration if heart rates fall below 60 bpm despite adequate ventilation (Perlman et al., 2015). While the requirement for CC is rare (0.1% of term and up to 15% of extremely preterm infants), the outcomes of infants requiring CC in the delivery room are devastating: 41–83% will die and 57–93% of survivors suffer moderate to severe disability (Barber and Wyckoff, 2006; Shah, 2009; Foglia et al., 2020).

Rapid restoration of cardiac function in asystolic infants is critical to restore blood flow and oxygen to the brain to minimize hypoxic injury. Currently, International Liaison Committee on Resuscitation (ILCOR) (Perlman et al., 2015) recommends CC to be initiated after umbilical cord clamping (UCC). However, this is associated with a delay in resuscitation onset caused by moving infants to a resuscitation table before CCs can start, which prolongs the period of hypoxia. Further, infants that survive CC have a high incidence of cerebral bleeding likely due to aggravated blood pressure fluctuations that cause rebound hypertension in the immediate post-asphyxial period (Bada, 2000; McCaul et al., 2006; Pinto et al., 2017). We have previously shown that a post-asphyxial overshoot in blood pressure can occur after return of spontaneous circulation (ROSC) in lambs undergoing immediate cord clamping (ICC), which increases the incidence of cerebral hemorrhage (Polglase et al., 2017). Therefore, an optimal CC strategy would rapidly restore cardiac function but avoid the overshoot in blood pressure and flow to the brain.

We have demonstrated that providing respiratory support and increasing pulmonary blood flow prior to UCC, described as physiological-based cord clamping (PBCC), does not impede the restoration in cardiac function in severely asphyxiated near-term lambs. Importantly, it does prevent the overshoot in blood pressure and flow to the brain, thereby reducing the incidence of cerebral hemorrhage (Polglase et al., 2017). However, it is unknown whether it is feasible to restore cardiac function in newborns requiring CC while attached to the umbilical cord. This is because diastolic pressures must increase above 15–20 mmHg before ROSC can be achieved (Paradis et al., 1990; Wyckoff, 2010), and it is possible that having the low resistance placental circulation in circuit reduces the increase in diastolic pressures during CCs. Further, it is unknown whether delaying UCC until after ROSC prevents the post-asphyxial hypertension seen upon restoration of cardiac function.

The aim of this study was to compare the cardiovascular response to CCs given prior to or after UCC in asystolic near-term lambs. We hypothesized that UCC after CC would increase the time to ROSC and reduce the post-asphyxial overshoot in blood pressure in asystolic near-term lambs.

## Materials and Methods

Experimental procedures were approved by Monash Medical Centre Animal Ethics Committee A, Monash University, Australia, and were conducted in accordance with the National Health and Medical Research Council of Australia’s guidelines.

### Instrumentation and Delivery

Pregnant Border-Leicester ewes (*Ovisaries*) at 139 ± 2 days gestation (mean ± SD; term ~148 days) were anesthetized by intravenous injection of thiopentone sodium (20 mg/kg; Jurox, NSW, Australia), followed by tracheal intubation and delivery of inhaled anesthesia (isofluorane 1.5–2.5% in oxygenated air; Bomac Animal Health, NSW, Australia). The fetal head and chest were exposed *via* hysterotomy for placement of an ultrasonic flow transducer of appropriate size (Transonic Systems, Ithaca, NY, USA) around the left main pulmonary artery, which was accessed *via* a left thoracotomy. A flow probe of appropriate size was placed around a carotid artery and a femoral artery, and catheters were inserted into a carotid artery and a jugular vein. Arterial pressures and blood flows were digitally recorded in real-time (1 kHz, Powerlab; ADInstruments, Castle Hill, NSW, Australia). After closure of the incisions in the neck and chest, the fetal trachea was intubated with a 4.5 mm cuffed endotracheal tube, and lung liquid was drained passively. A transcutaneous arterial oxygen saturation (SpO_2_) probe (Masimo, Radical 4, CA, USA) was placed around the right forelimb of the lamb, and the output was digitally recorded. A near infrared spectroscopy (NIRS) optode (Casmed Foresight, CAS Medical Systems Inc., Branford, CT, USA) was placed over the left frontal cortex and used to continuously measure cerebral tissue oxygen saturation (SctO_2_). Cerebral oxygen extraction was calculated as SaO_2_-SctO_2_/SaO_2_ (Polglase et al., 2015).

The fetus was removed from the uterus, dried and placed on the ewes’ abdomen on a hot water bottle to maintain core body temperature. Additional flow probes (4 mm) were placed around an umbilical artery and vein. Instantaneous blood flows in the left pulmonary blood flow (pulmonary blood flow, PBF) carotid artery (carotid arterial blood flow, CaBF), and umbilical artery and vein were recorded digitally using a data acquisition system (Powerlab; ADInstruments, Castle Hill, NSW, Australia). CaBF has been shown to strongly correlate with cerebral blood flow in lambs (van Bel et al., 1994; Covert et al., 1996). Arterial pressures were measured using pressure transducers (PD10; DTX Plus Transducer; Becton Dickinson, Singapore) and were also recorded digitally as airway pressures, tidal volumes, and cerebral tissue and preductal oxygenation.

Asphyxia was induced by clamping of the umbilical cord for lambs undergoing ICC or occlusion of the maternal internal iliac artery for lambs undergoing PBCC as described previously (Hooper et al., 1995; Supramaniam et al., 2004); this reduces uterine perfusion without interfering with umbilical blood flow. Asphyxia was continued until asystole was obtained, classified by the absence of discernible activity on the blood pressure/ECG trace. Lambs were then allocated to either:

1. ICC (*n* = 12): the cord was clamped and CPR was initiated within 30 s;
2. PBCC, whereupon ventilation was commenced, and CPR was initiated while the lamb was still attached to the umbilical cord with UCC occurring 1 min after ROSC (PBCC_1_; *n* = 8); or
3. PBCC whereupon ventilation was commenced, and CPR was initiated while the lamb was still attached to the umbilical cord with UCC occurring 10 min after ROSC (PBCC_10_; *n* = 8).

CPR was initiated with a 30 s sustained inflation to 30 cm H_2_O using 100% O_2_, followed by positive pressure ventilation using a T-piece device (Neopuff; Fisher and Paykel Healthcare, Auckland, New Zealand) with a peak inflation pressure of 30 cm H_2_O and end-expiratory pressure of 5 cm H_2_O targeting 60 bpm. One minute after ventilation, onset CCs were initiated using an asynchronous technique at a 3:1 ratio. Epinephrine (0.1 mg/kg bodyweight) was given intravenously 1 min after CCs were initiated, and every 3 min thereafter for a maximum of three doses. Upon ROSC, lambs were transferred to volume guaranteed ventilation targeting 7 ml/kg with warm humidified air (Dräeger Babylog 8000+, Dräeger, Lübeck, Germany). The fraction of inspired oxygen was adjusted to maintain arterial oxygen saturation between 90 and 95%, and ventilation parameters were adjusted to maintain PaCO_2_ between 45 and 55 mmHg. Upon ROSC, lambs were sedated (Alfaxan i.v. 5–15 mg/kg/h in 5% dextrose; Jurox, NSW, Australia) to prevent spontaneous breathing. Blood samples were collected regularly *via* the carotid artery catheter, and blood gas parameters were measured using a blood gas analyzer (ABL30, Radiometer, Copenhagen, Denmark) to monitor the lamb’s well-being and guide respiratory support.

### Statistical Analysis

All baseline fetal and physiological data were compared using a one-way ANOVA (Graphpad Prism; GraphPad Software, CA, USA). Two-way repeated measures ANOVA with Holm-Sidak *post hoc* comparison was used to compare serial physiological data (SigmaPlot; Systat Software Inc., CA, USA). Statistical significance was accepted at *p* < 0.05.

## Results

### Fetal Characteristics

**Figure 1:**
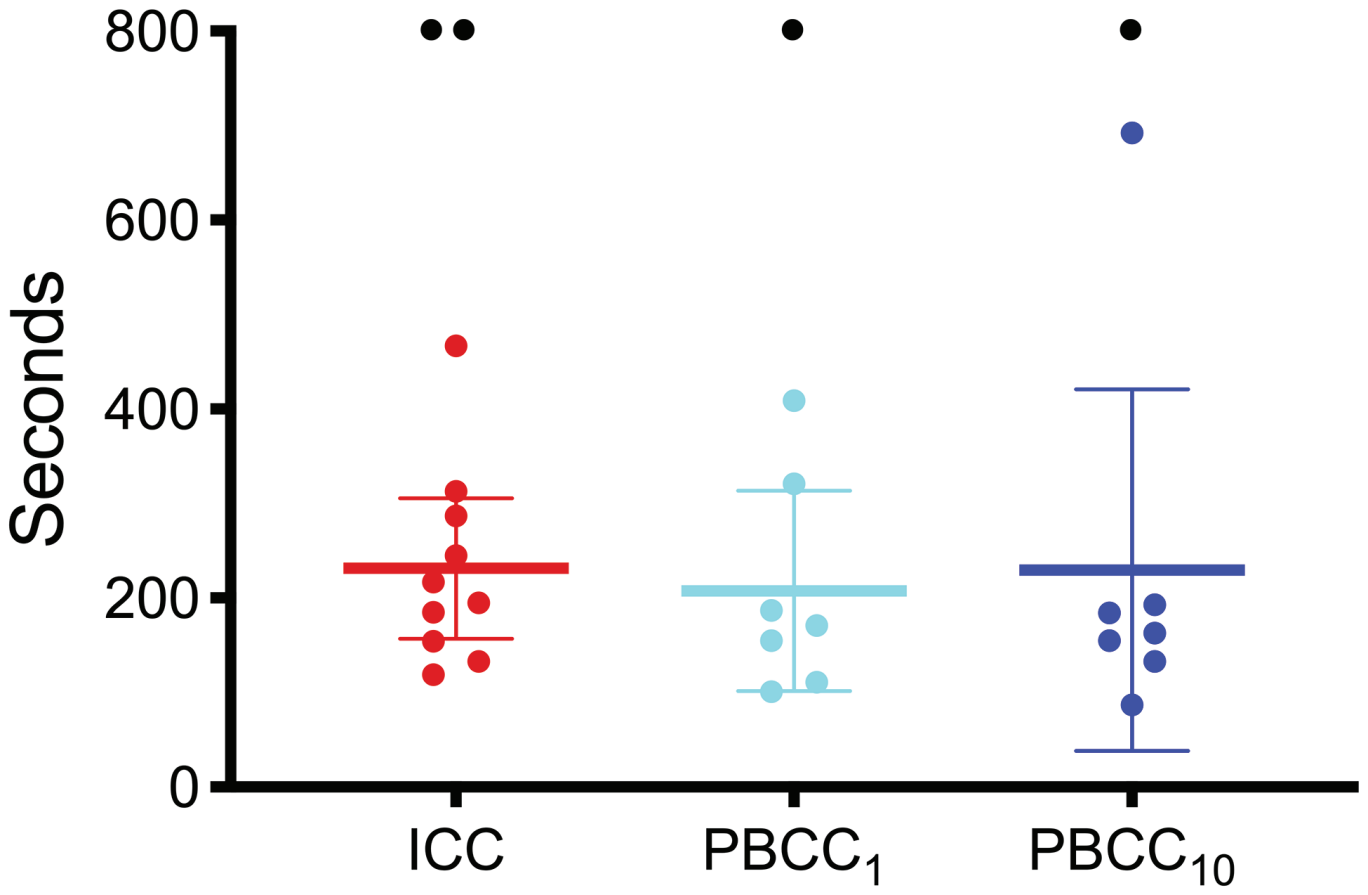
Time to return of spontaneous circulation in immediate cord clamping (ICC; red), Physiologically-based cord clamping, PBCC_1_ (light blue) and PBCC_10_ (dark blue) lambs. Individual animals are shown with mean (95% CI) included.

Fetal characteristics are outlined in Table 1. There were no differences between groups in any blood gas or physiological variable prior to induction of asphyxia. The mean duration of asphyxia for all lambs was 15.4 ± 3.8 min with no differences in duration of asphyxia between the groups. Two lambs in the ICC group and one each in the PBCC_1_ and PBCC_10_ groups failed to reach ROSC during CPR, meaning that the final analysis was conducted on *n* = 10 ICC lambs and *n* = 7 PBCC_1_ and *n* = 7 PBCC_10_ lambs. There was no statistical difference in failure to reach ROSC between groups. There were no differences in the time taken for ROSC between groups (Figure 1): mean (range) ICC: 231 s (119–467 s), PBCC_1_: 208 s (101–409 s), and PBCC_10_: 230 s (87–692 s).

**Table 1:** Fetal characteristics.

	ICC	PBCC_early_	PBCC_late_
*n*	10	7	7
Birth weight (kg)	4.1 ± 0.5	5.1 ± 0.7	5.28 ± 0.2
Males *n*, (%)	6 (60)	4 (57)	4 (57%)
pH	7.23 ± 0.06	7.24 ± 0.06	7.23 ± 0.06
PaCO_2_ (mmHg)	57.2 ± 9.7	60.7 ± 10.1	64.6 ± 9.2
PaO_2_ (mmHg)	18.6 ± 5.5	20.0 ± 6.0	21.7 ± 5.7

Values are mean ± SD.

### Blood Gas Measurements and Oxygenation

**Figure 2:**
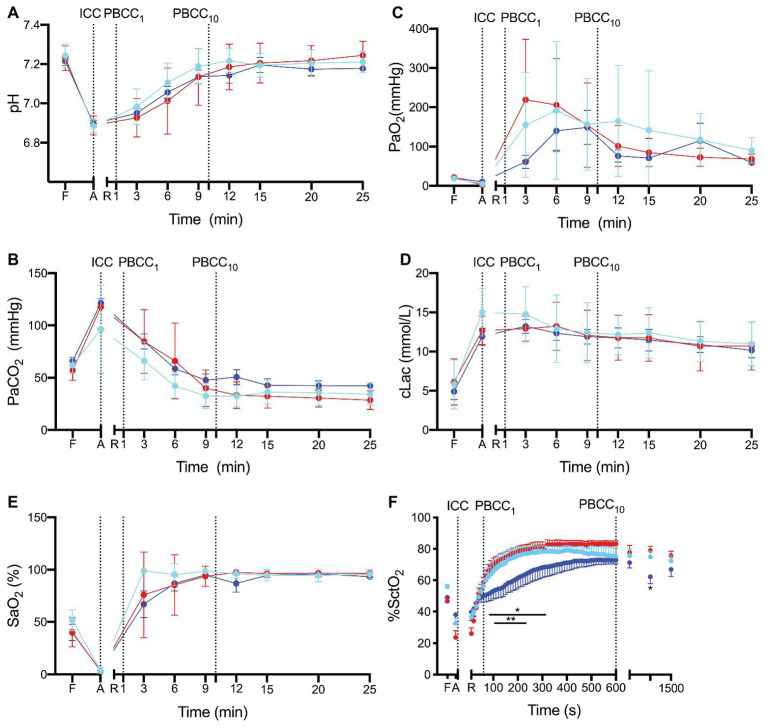
**(A)** pH, **(B)** the partial arterial pressure (Pa) of carbon dioxide (CO_2_), **(C)** Pa of oxygen (O_2_), **(D)** lactate, **(E)** arterial saturation SaO_2_ and **(F)** cerebral oxygenation (SctO_2_) measured prior to delivery (fetal, F), at the end of the asphyxial period (A) and upon return of spontaneous circulation (R) in ICC (red), PBCC_1_ (light blue), and PBCC_10_ (dark blue) lambs. Data are mean ± SD.

pH, PaCO_2_, PaO_2_, SaO_2_, and lactate (Figures 2A–E) and base excess (data not shown) were not different between groups prior to the study (fetal controls levels), at end asphyxia (indicating similar apshyxial levels between groups) or upon ROSC. Cerebral oxygenation was significantly lower in PBCC_10_ compared to ICC from 80 to 320 s and at 15 min, and compared to PBCC_1_ from 120 to 220 s (Figure 2F).

### Return of Spontaneous Circulation: Physiology

#### Umbilical Blood Flow

**Figure 3:**
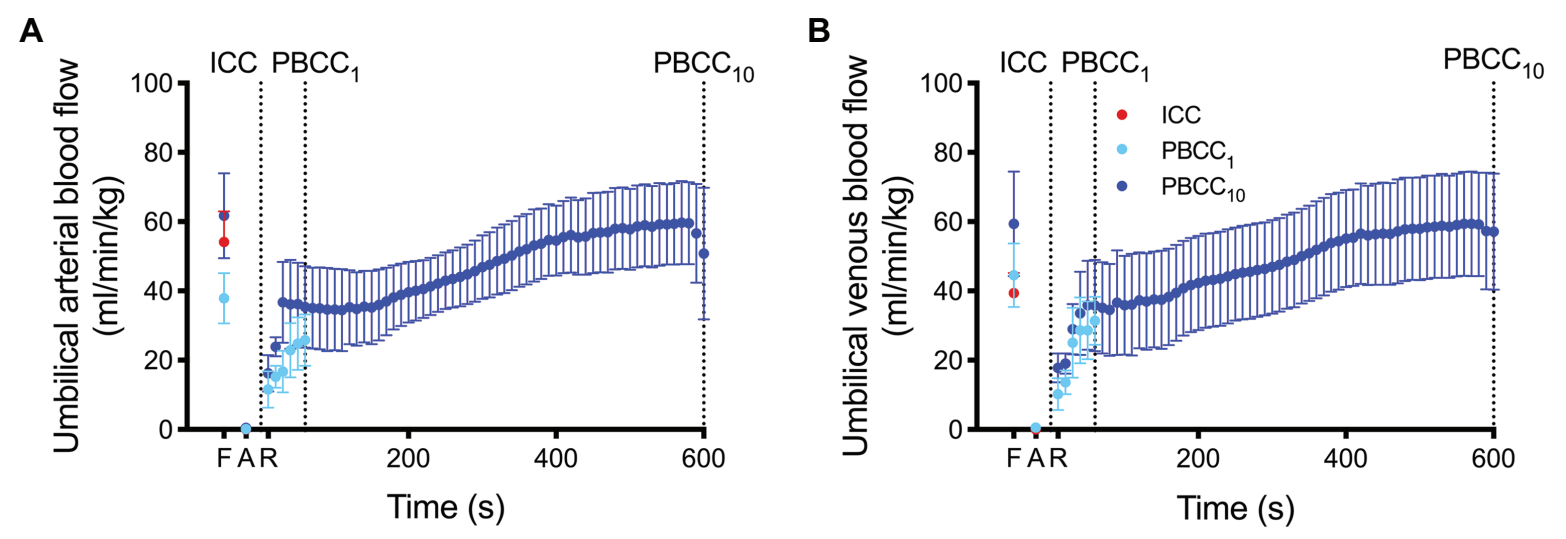
**(A)** Umbilical arterial blood flow (UaBF) and **(B)** umbilical venous blood flow (UvBF) in ICC (red) and PBCC_10_ lambs (dark blue) measured prior to asphyxia (fetal), at end asphyxia, and upon return of spontaneous circulation. Umbilical venous and arterial blood flow ceases in the ICC upon cord clamping. Data are mean ± SD.

Umbilical arterial and venous blood flow are shown in Figure 3. Umbilical blood flows were reduced to ~0 ml/min/kg at end asphyxia, but were not significantly different from fetal values at 90 s and were restored to absolute fetal values within ~400 s of ROSC within the PBCC_10_ group.

**Figure 4:**
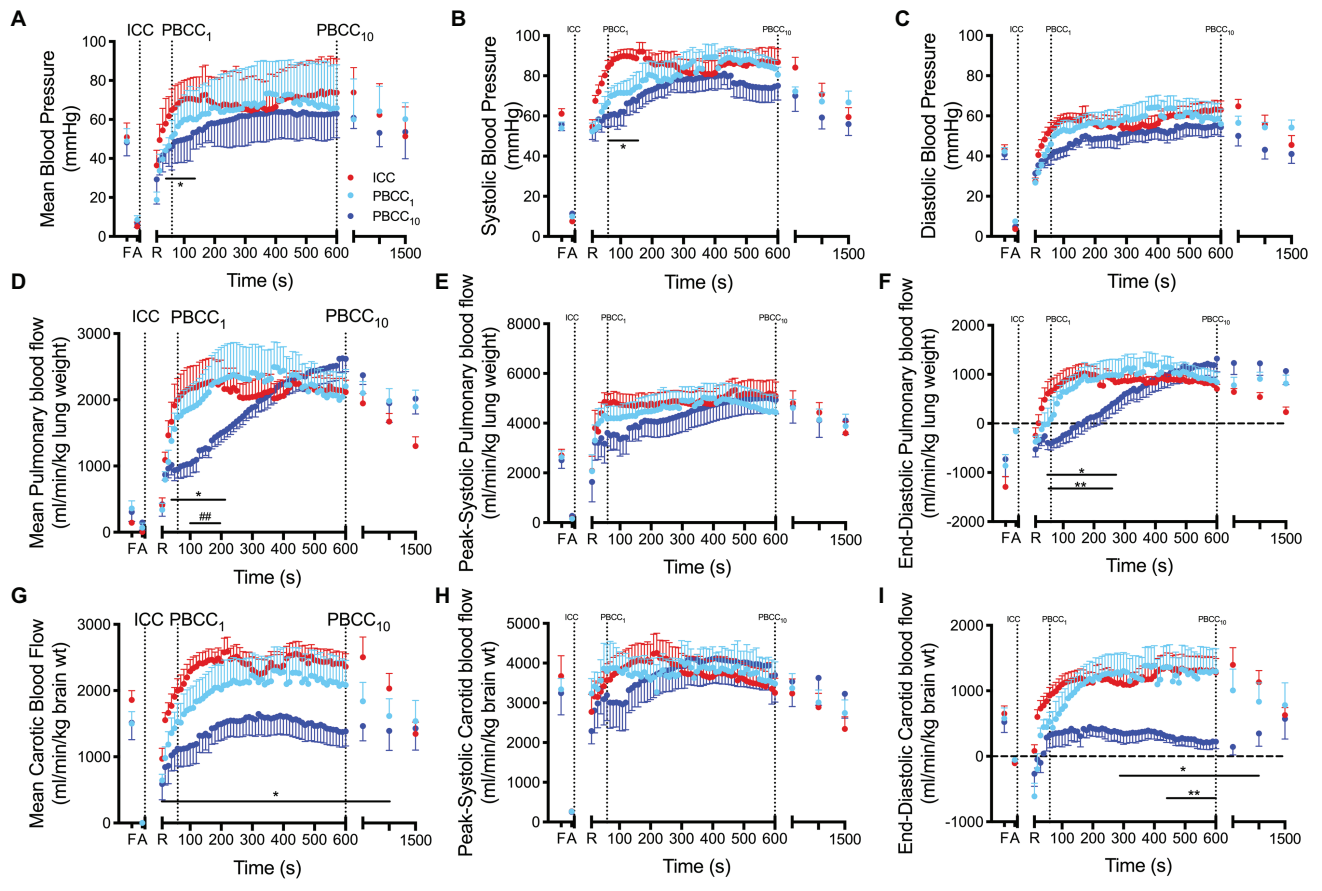
**(A–C)** Mean, systolic and diastolic arterial blood pressure, **(D–F)** mean, peak-systolic and end-diastolic pulmonary blood flow and **(G–I)** mean, peak-systolic and end-diastolic carotid arterial blood flow measured prior to delivery (fetal, F), and at the end of the asphyxial period (A), and upon spontaneous return of circulation (R) in ICC (red), PBCC_1_ (light blue), and PBCC_10_ (dark blue) lambs. ^*^indicates significant difference (*p* < 0.05) between PBCC_1_ and ICC lambs at that time point. ^**^indicates significant difference (*p* < 0.05) between PBCC_1_ and PBCC_10_ lambs at that time point. ^##^indicates trend (*p* < 0.07) between PBCC_1_ and PBCC_10_ lambs at that time point. Data are mean ± SD.

#### Blood Pressure and Heart Rate

Mean blood pressure was significantly lower in PBCC_10_ compared to ICC lambs from 50 to 160 s but was not different thereafter (Figure 4). Peak mean blood pressures for each group were ICC: 74.6 mmHg, PBCC_1_: 73.6 mmHg, and PBCC_10_: 64.0 mmHg. Systolic blood pressure was significantly lower in PBCC_10_ compared to ICC lambs from 60 to 180 s but was not different thereafter. Mean or systolic blood pressure was not different between PBCC_1_ lambs and either group. Diastolic pressure was not different between groups. Heart rate was not different between groups (data not shown).

#### Pulmonary Blood Flow

Mean PBF was significantly lower in PBCC_10_ compared to ICC from 50 to 200 s and at 15 min and tended to be lower (*p* < 0.07) compared to PBCC_1_ from 100 to 200 s (Figure 4). End-diastolic PBF was significantly lower in PBCC_10_ compared to ICC from 30 to 270 s and at 15 min and compared to PBCC_1_ from 40 to 260 s (Figure 4). No difference was observed between PBCC_1_ and ICC lambs. Peak systolic PBF was not different between groups.

#### Carotid Blood Flow

Mean carotid blood flow was significantly lower in PBCC_10_ compared to ICC lambs after ROSC until 20 min (group difference *p* = 0.03; Figure 4). PBCC_1_ lambs were not different to ICC or PBCC_10_ lambs. End-diastolic carotid blood flow was significantly lower in PBCC_10_ compared to ICC lambs (overall group difference *p* = 0.02) from 5 to 15 min and to PBCC_1_ lambs (overall group difference *p* = 0.06) from 440 s to 10 min. Peak systolic carotid blood flow was not different between groups. PBCC_1_ and ICC were not different from each other.

## Discussion

Current ILCOR guidelines (Perlman et al., 2015) recommend immediate UCC for infants requiring CC. In this study, we aimed to determine whether there was any benefit of providing ventilation and CC prior to UCC compared to the current clinical guidelines. The main findings of this study are (1) CC is possible with an intact umbilical cord, and no obvious adverse effects were noted and (2) if UCC was delayed for 10 min after ROSC, we found significant reductions in the post-asphyxial hypertension, cerebral blood flow, and cerebral oxygenation compared to immediate ICC and PBCC_1_. These findings highlight the critical importance that the timing of UCC relative to ROSC can have on physiological outcomes in severely asphyxiated newborns.

Our primary goal was to determine whether it was feasible to undertake CC prior to UCC. We hypothesized that CCs could be ineffective with an open placental circuit due to an inability to generate enough end-diastolic pressure (15–20 mmHg; Paradis et al., 1990; Wyckoff, 2010) to restore cardiovascular function. Contrary to this, we found no differences in diastolic pressures between a clamped and an open umbilical cord during CCs, or upon ROSC. Additionally, we found no differences in the ability to obtain ROSC and also the time that it took to achieve ROSC between ICC and PBCC groups. This is an important finding. It dispels the perception that the generation of adequate end-diastolic pressures during chest compressions are not possible if conducted during delayed cord clamping, and proves that outcomes following PBCC were not different to the current clinical standard. This is evidenced by the similar duration of CC and number of epinephrine doses received (1–2 doses per lamb) between groups.

We have previously shown that ICC before lung aeration exposes the asphyxic newborn to a rapid overshoot in blood pressure, resulting in damage to the cerebral micro-vessels (Polglase et al., 2017). The explanation for this is that the cerebral resistance vessels are maximally vasodilated in response to the asphyxia to maximize flow and oxygen delivery to the brain. As cerebral autoregulation of blood flow is temporarily lost, cerebral blood flow is pressure passive (Herrera-Marschitz et al., 2015), and so any sudden large increases in blood pressure exposes the delicate micro-vessels (that are normally protected from high pressures) to high pressures and flows. This causes microvascular leakages, compromises blood brain barrier integrity, and increases the risk of hemorrhage (Lou et al., 1979). We have previously shown that ventilation of mildly asphyxiated newborns before UCC mitigates the post-asphyxia overshoot in blood pressure and reduces cerebrovascular injury (Polglase et al., 2017). As such, in this study, we postulated that PBCC would have the same effect, but only if the PBCC extended throughout the sympathetic rebound response. Indeed, we found that UCC 1 min after ROSC did not reduce/prevent the post-asphyxia hypertension and actually had a very similar profile to ICC. While UCC at this time did not prevent the overshoot, it did not worsen immediate outcomes relative to ICC lambs.

The rebound tachycardia and hypertension is a common feature of post-asphyxial episodes before and after birth, even in the absence of exogenous epinephrine (Bennet et al., 1999; Wassink et al., 2007). It is most likely driven by a large increase in sympathetic autonomic activity (akin to the “fight or flight” response), which increases sympathetic drive to the heart and the release of epinephrine from the adrenal medulla (Jones et al., 1988; Hooper et al., 1990). In adults following a myocardial infarction (MI) that causes a similar loss in cardiac function, the post MI increase in sympathetic drive is associated with ventricular arrhythmias, sudden heart failure, and an increased risk of death (Jardine et al., 2005). We commonly observed post-asphyxial arrhythmias in our lambs, and so it is possible that this increase in sympathetic drive is as harmful to the newborn as it is to the adult but may manifest in a different way. That is, the rebound hypertension contributes to cerebral vasculature injury, and it is possible that the exogenous epinephrine, which is necessary to achieve ROSC, further contributes to this injury. Indeed, while epinephrine has much greater activity on beta adrenoceptors, in very high doses, it also activates alpha adrenoceptors. As alpha adrenoceptors predominate in peripheral vascular beds, in high doses, epinephrine not only increases cardiac output and heart rate, but it also causes vascular vasoconstriction, and thereby greatly increases blood pressure.

Studies in adult rats with asphyxia-induced cardiac arrest found that while epinephrine administration improved the speed of return of cardiac function, it increased post-asphyxia hypertension and tachycardia, resulting in increased mortality compared to saline controls (McCaul et al., 2006). Studies in preterm infants receiving CC in the delivery room also found aggravated pressure fluctuations and increased intraventricular hemorrhage (IVH) after epinephrine administration (Bada, 2000). Thus, while epinephrine administration is essential for ROSC in our lambs, it may increase the risk of poor cerebral outcomes. Intravenous administration of epinephrine in asystolic lambs results in a peak plasma concentration of adrenalin within 1 min (Vali et al., 2017a). Although not measured in this study, it may be possible that clamping the UCC while the physiological effects of exogenous epinephrine are still present may contribute to the hypertensive overshoot we observed.

When PBCC occurred for 10 min after UCC, the post-asphyxia overshoot in cerebral blood flow and cerebral oxygenation was also significantly reduced. The reason for the reduction in cerebral perfusion is due to the presence of a patent placental circuit. With aeration of the lung and clamping of the umbilical cord, 100% of right ventricular output is directed toward the lungs, as well as a considerable proportion of left ventricular output *via* a reversal of blood flow across the ductus arteriosus, from right-to-left to left-to-right shunting (Crossley et al., 2009; Hooper et al., 2015a,b). As indicated by end-diastolic PBF, left-to-right shunting was not established until 3–4 min in the PBCC_10_ lambs as systemic blood flow was preferentially flowing toward the placenta. In essence, the placenta worked as a pressure relief valve, providing an alternative route for blood to flow, thus greatly reducing overall vascular resistance. Importantly, it appears that the timing of UCC after ROSC is critically important for improving physiological stability, particularly blood pressures, flows, and oxygen delivery to the brain. A clearer understanding of the mechanisms/pathways driving the overshoot in blood pressure leading to the increase in cerebral perfusion may predict an optimal timing for UCC after ROSC.

Finally, similar to previous studies (Vali et al., 2017a,b), we found a significant overshoot in cerebral oxygenation (as indicated by NIRS) in both the ICC and PBCC_1_ groups compared to the PBCC_10_, despite rapidly decreasing FiO_2_ immediately after ROSC. This raises critical questions about the potential risk of oxidative stress and free radical generation when a high FiO_2_ (100%) is used during CPR (Vento et al., 2012; Saugstad, 2013). Animal studies have repeatedly shown that initiating CPR with lower FiO_2_ (usually 21%) results in no difference in the rate or speed of ROSC (Solevag et al., 2010a,b, 2016). However, interestingly, a recent study found no difference in oxidative stress or white matter inflammation when CPR was initiated with 18, 21, or 100% (Solevag et al., 2020), suggesting that reducing oxidative stress in the brain is likely to be complex in the setting of asphyxia.

## Limitations

It is important to emphasize that this was a physiological study conducted in near-term lambs designed to specifically investigate the physiology of the transition at birth when complicated by birth asphyxia. Extrapolating to the clinical situation, or even initiating studies in humans, needs to be done with caution. Indeed, conducting CCs on an intact umbilical cord is technically challenging, and it is unlikely that maintaining an asystolic newborn on an umbilical cord for 10 min after ROSC is feasible with current technology, although purpose built resuscitation tables may be a solution to this problem (Brouwer et al., 2018). Further, our findings raise the concern that initiating interventions in an asphyxiated infant before we fully understand the physiology could be catastrophic. Indeed, while simply delaying cord clamping for 1 min after ROSC appeared to cause no harm, the potential to suddenly increase peripheral vascular resistance during the rebound response is potentially hazardous. It should also be noted that we only observed the lambs for 20 min, after ROSC, so while no adverse physiological effects were noted, particularly within the PBCC_10_ group, studies of longer duration would be needed to confirm this assertion. The lambs were anesthetized throughout the study, which prevents spontaneous breathing. While we know that gasping during asphyxia does not initiate the physiological transition at birth (Ong et al., 2016), the physiological response after ROSC in spontaneously breathing models is not yet known. The method of inducing asphyxia was different between the groups. This was done for ethical reasons related to reducing the overall number of ewes required in the study by allowing us to use twin pregnancies. However, the method for asphyxia in this study does not alter the findings, given that the study focuses on the return of circulation after asphyxia and not the response to asphyxia *per se*.

In conclusion, we found that initiating CCs prior to UCC results in a similar restoration of cardiac function as to the current standard of initiating CCs after UCC. Delaying UCC until 1 min after ROSC had no physiological benefit compared to the current clinical standard; however, delaying UCC until 10 min after ROSC had profound benefits on cerebral pressures, flows, and oxygenation. Therefore, the timing of UCC after ROSC is complex, but understanding the physiology is critical if PBCC is to be attempted in asphyxiated newborns.

## Data Availability Statement

The raw data supporting the conclusions of this article will be made available by the authors, without undue reservation.

## Ethics Statement

The animal study was reviewed and approved by Monash Medical Centre Animal Ethics Committee, Monash University, Australia.

## Author Contributions

GP contributed to the conceptualization, the methodology, the validation, the formal analysis, the investigation, the writing, the supervision, the funding acquisition, and the project administration. GS contributed to the conceptualization, the visualization, the methodology, the investigation, the writing, the supervision, and the funding acquisition. CR, DB, and SB contributed to the methodology, the investigation, the writing, and the supervision. SM contributed to the methodology, the investigation, the funding acquisition, the writing, and the supervision. VS, KC, and RG contributed to the methodology, the resources, the investigation, the writing, and the supervision. MK and AG contributed to the conceptualization, the methodology, the investigation, the writing, and the supervision. SH contributed to the conceptualization, the methodology, the validation, formal analysis, the investigation, the writing, the supervision, and the project administration. All authors contributed to the article and approved the submitted version.

### Conflict of Interest

The authors declare that the research was conducted in the absence of any commercial or financial relationships that could be construed as a potential conflict of interest.
